# Identification and Aggressiveness of *Fusarium* Species Associated with Onion Bulb (*Allium cepa* L.) during Storage

**DOI:** 10.3390/jof10020161

**Published:** 2024-02-19

**Authors:** Roderic Gilles Claret Diabankana, Mikhail Frolov, Bakhtiyar Islamov, Elena Shulga, Maria Nikolaevna Filimonova, Daniel Mawuena Afordoanyi, Shamil Validov

**Affiliations:** 1 Laboratory of Molecular Genetics and Microbiology Methods, Kazan Scientific Center of the Russian Academy of Sciences, 420111 Kazan, Russia; 2 Academic and Research Centre, Institute of Fundamental Medicine and Biology, Kazan Federal University, 420008 Kazan, Russia

**Keywords:** biocontrol agent, *Fusarium* species complex, pathogenicity assay

## Abstract

Plant pathogens present a major challenge to crop production, leading to decreased yield and quality during growth and storage. During long-term storage, healthy onions can develop diseases from latent pathogen infections. This poses a challenge for onion growers because infected bulbs without visible symptoms can lead to significant crop losses during the growing season. In this study, we aimed to isolate and identify *Fusarium* species from yellow onion bulbs (*Allium cepa* L.) that developed disease symptoms during storage. The aggressiveness of these strains against onion bulbs and seedlings was also evaluated. The isolated strains were further subjected to morphological and molecular differentiation. The results revealed that all 16 isolated strains belonged to the *Fusarium* complex species *incarnatum-equiseti* and *Fusarium fujikuroi*, namely, *F. proliferatum* (98%), *F. oxysporum* (1%), and *Fusarium* sp. (1%). Koch’s postulate analysis of isolated strains revealed varying aggressiveness on onion bulbs and plants depending on fungal species. Disease symptoms developed more slowly on plants than on onion bulb plants according to Koch’s postulates. Moreover, the results revealed that *Fusarium* strains that can infect onion plants were less pathogenic to onion bulbs and vice versa. In addition, three isolates were found to be non-pathogenic to onions. Furthermore, the in vitro control of *Fusarium* species through *Bacillus velezensis* KS04-AU and *Streptomyces albidoflavus* MGMM6 showed high potential for controlling the growth of these pathogenic fungi. These results may contribute to the development of environmentally friendly approaches for controlling onion spoilage caused by pathogens during storage.

## 1. Introduction

Technological advancements in agriculture have greatly contributed to the development of global crop production. However, despite the progress made in production, postharvest loss during storage remains a significant challenge. The spoilage of crops caused by microorganisms, including many bacterial and fungal species, has emerged as a critical issue in modern agriculture, resulting in substantial economic losses and threats to worldwide food security [1]. According to the Food Safety Department of the World Health Organization, foodborne illness affects nearly 1 out of 10 individuals worldwide, resulting in approximately 420,000 deaths each year and a significant loss of 33 million years of healthy life. The onion bulb (*Allium cepa* L.) is an important seasonal crop with a high commercial value, and its yield is affected by several factors during long-term storage. Onion bulbs are susceptible to numerous postharvest diseases caused by several phytopathogens from genera such as *Aspergillus*, *Fusarium*, *Rhizopus*, *Penicillium*, *Urocystis*, and *Alternaria* [1,2]. The postharvest losses of onions can reach as high as 40–60% [3,4,5]. Among these losses, 10–40% can be attributed to diseases caused by fungi, bacteria, nematodes, or viruses [6,7]. Among phytopathogenic microorganisms, *Fusarium* is the most persistent soilborne fungal plant pathogen [8,9]. Species of this genus are prevalent in soils across diverse climate regions and are associated with a wide variety of plants, causing considerable damage to crops [10,11]. *Fusarium*, a major causal agent of bulb basal rot, has been reported in several studies [12,13,14]. *Fusarium* pathogens, known for their devastating effects on plant health, can pose significant risks to human health [15]. These pathogens not only infect plants but also produce toxic secondary metabolites called mycotoxins, which can contaminate food and feed products. The consumption of such contaminated food can lead to severe health issues in humans, including acute poisoning, chronic diseases, and even cancer [16]. Furthermore, *Fusarium* species can also cause opportunistic infections in immunocompromised individuals, such as those with compromised immune systems or those recovering from surgery [17]. These infections can manifest in various forms, including skin infections, keratitis (eye infections), and invasive disseminated diseases [18]. Control of postharvest onion diseases is essential to ensure the long-term storage and marketability of onion bulbs. Several methods such as sanitation and hygiene practices, temperature and humidity management, as well as chemical treatments, have been used to control the development of disease during long-term onion bulb storage [19,20,21]. These methods offer effective disease management, however, they are also supplied with challenges related to their cost, potentially raising concerns regarding food safety and environmental impact, as well as the risk of resistance development [22]. Onion bulbs affected by phytopathogens may not show symptoms of infection during visual inspection (a method that is usually used at onion packaging enterprises). However, over time, these pathogens may cause severe damage during storage [23]. In addition, these infected onion bulbs without obvious symptoms could reach markets nationwide. This concern is exacerbated by the presence of known mycotoxins released by these pathogens, which may negatively affect human health [24,25]. Hence, identifying phytopathogenic microorganisms responsible for onion crop spoilage and determining their sources are crucial for making decisions about plant protection measures. Biological control agents (BCAs) are a promising alternative for controlling plant pathogens, offering environmentally friendly and sustainable methods of suppressing diseases [26]. Several BCAs have shown efficacy against phytopathogenic microorganisms belonging to the following genera *Fusarium*, *Rhizopus*, *Penicillium*, *Alternaria Pantoea*, *Pseudomonas*, *Burkholderia*, *Xanthomonas*, and *Erwinia* [27,28]. These BCAs act by competing with pathogens for nutrients and niches, as well as, by producing antifungal compounds [29,30]. In this study, we focused on identifying and characterizing spoilage causal agents, specifically *Fusarium*, that affect the most common variety of yellow onions (*Allium cepa* L.) sold in various commercial marketplaces in Kazan (Tatarstan, Russia), as well as on the development of an environmentally friendly approach to control these causal agents.

## 2. Materials and Methods

### 2.1. Isolation and Identification of Pathogens from Diseased Onion Bulbs

**Figure 1:**
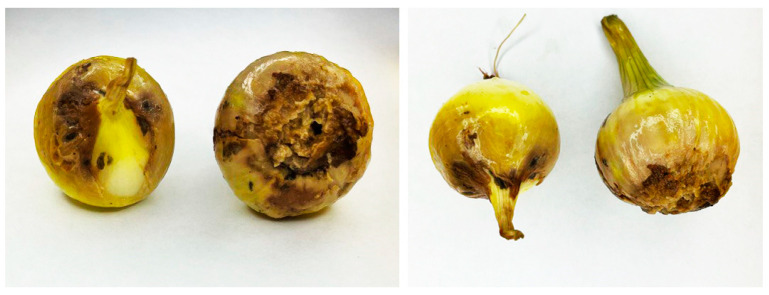
The infected yellow onion bulbs were used in this study.

The onion bulbs (10 pieces of bulbs) variety “golden semko” (Semko-Junior LLC, Moscow, Russia) used in this study were purchased from different commercial marketplaces located in Kazan (Tatarstan, Russia,) and stored at room temperature and in the absence of light. During storage, onion bulbs with rotting parts were randomly removed and used to identify the causal pathogens of *Fusarium* species (Figure 1).

For this purpose, the tissues were cut into small segments, surface disinfected with a 70% ethanol solution, and rinsed with sterile water. The segments were then immersed for 1 min in a 2% sodium hypochlorite solution (containing 0.5% sodium dodecyl sulfate (SDS)), followed by washing with distilled water. The segments were plated on potato dextrose agar (HIMEDIA, Moscow, Russia) supplemented with rifampicin to a final concentration of 100 µg/mL and incubated at 27 ± 1 °C in the dark. The grown fungi were preselected based on their morphology and replated onto potato dextrose agar (PDA). Furthermore, to obtain a pure fungal colony, the single-spore subculture method was performed according to Leslie and Summerell [31]. After serial dilution, aliquots of 100 µL from the last three dilutions (5-fold dilution) were plated on Sabouraud Dextrose Agar (HIMEDIA, Moscow, Russia) and incubated at 27 ± 1 °C. To distinguish the isolated fungal species, the grown colonies were fingerprinted using BOX-PCR. The selected strains were further subjected to molecular sequence-based identification.

### 2.2. Molecular Analysis

#### 2.2.1. DNA Isolation

Chromosomal DNA was extracted from 5-day-old fungi grown on PDA agar plates using a phenol–chloroform extraction method according to Green et al. [32]. The quantification of isolated DNA was analyzed using a NanoDrop spectrophotometer and gel electrophoresis [33].

#### 2.2.2. DNA Fingerprinting Analysis (BOX-PCR)

BOX-PCR was performed in a 25 µL volume containing 2.5 µL of 10× PCR buffer, 0.4 µL of dNTP mixture (10 µM), 1.25 µL of 10 µM BOXAIR (5′-CTACGGCAAGGCGACGCTGACG-3′), 5.0 µL of DNA template (50 ng), 1.0 µL of Taq DNA polymerase (5 U), 50 ng of template DNA, and free nuclease-free water. Amplification was performed using a T100 thermocycler (Bio-Rad, Hercules, CA, USA) under the following conditions: initial denaturation at 95 °C for 2 min; 30 cycles of 94 °C for 30 s and 58 °C for 30 s; and 72 °C for 8 min. The final cycle was extended to 72 °C for 10 min. The DNA fragments were detected via 1.5% agarose gel electrophoresis and visualized on Gel Doc EZ Imager with Image Lab 6.0 software (Bio-Rad, Hercules, CA, USA). Dendrogram analysis was performed using GelJ software v.3.0 (Java Application, Logroño, Spain). Hierarchical clustering analysis of the BOX DNA profiles of the isolated fungi was performed using the Jaccard similarity coefficient and UPGMA clustering analysis methods, with the matching band tolerance set at 1%.

#### 2.2.3. Molecular Fungal Species Identification and Phylogenetic Relationships

The preselected fungal strains were molecularly identified via PCR amplification and fragmentation sequencing analysis of the targeting translation elongation factor-1 alpha (tef-1α) gene and the universal internal transcribed spacer (ITS). The polymerase chain reaction amplification was performed using QuantStudio 5 (Thermo Fisher Scientific, Cleveland, OH, USA) in a 50 µL reaction mixture containing 1 µL of each (100 µM) primer, 10 mM DNTP mixture, 10 µL of PCR master mix (Evrogen, Moscow, Russia), 3 µL of template DNA (50 ng/mL), and water-free nuclease. For tef-1α (F-5′-ATGGGTAAGGAAGACAAGAC-3′; R-5′-GGAAGTACCAGTGATCATGTT-3′) [34]. PCR amplification was performed under the following conditions: 95 °C for 2 min; 30 cycles of 94 °C for 30 s, 47 °C for 20 s, and 72 °C for 30 s. The final PCR cycle was followed by a cycle at 4°C for 10 min. The universal primer (ITS1-F-5′-TCCGTAGGTGAACCTGCGG-3′; ITS4-R-5′-GCTGCGTTCTCCATCGATGC-3′) was used to amplify the entire ITS region [35]. The amplification was performed under the following conditions: denaturation at 95 °C for 5 min; 35 cycles of 95 °C for 50 s, 56 °C for 50 s, and 72 °C for 1 min; and a final cycle of 72 °C for 10 min. The obtained PCR products were fragmented by electrophoresis in 1.5% agarose gel and visualized on Gel Doc EZ Imager with Image Lab 6.0 software (Bio-Rad, Hercules, CA, USA). The target fragment was purified using a DNA cleanup kit (Evrogen, Moscow, Russian) according to the manufacturer’s protocol. After purification, the DNA fragment was sequenced by the Sanger method using an automated sequencer 3500×L Dx Genetic Analyzer (Applied Biosystems, Waltham, MA, USA). The chromatograms were further analyzed using Snapgene software v. 7.0 (GSL BioTech LLC, San Diego, CA, USA).

The generated partial fragments were further compared with fungal DNA sequences using an online approach against the *Fusarium* multilocus sequence (MLST) database (https://fusarium.mycobank.org/page/Fusarium_identification; 20 September 2023). Furthermore, the obtained consensus sequences were subjected to BLAST searches for homology against the GenBank database using the BLASTN tool [36]. More than 40 related trains (with less than 96% similarity) were downloaded from NCBI and used to construct a phylogenetic tree. The bootstrap consensus tree inferred from 1000 replicates was used to represent the evolutionary history of the taxa analyzed [37]. Branches corresponding to partitions reproduced in less than 50% of the bootstrap clustering threshold were generated. Evolutionary distances were computed using the maximum composite likelihood method [38] and are expressed in units of the number of base substitutions per site. All ambiguous positions were removed for each sequence pair (pairwise deletion option). Evolutionary analyses were performed using MEGA11 [39].

### 2.3. Test of Pathogenicity

#### 2.3.1. Preparation of Conidial Suspension of Isolated Fungal Strains

The conidial suspension of each isolated fungal strain was prepared from fungal isolated cultures grown up to seven days in Sabouraud broth (SDA; Merck, Darmstadt, Germany) at 25 ± 1 °C with an agitation rate of 150 rpm. After sporulation, the culture was passed through sterile cotton wool for spore filtration. The colony forming unit (CFU) was measured using a hemocytometer.

#### 2.3.2. Pathogenicity Assay of Onion Seeds

The pathogenicity of the isolated fungus was tested in a pot containing sterile sand supplemented with a plant nutrient solution. For this purpose, the onion seeds variety “golden semko” (Semko-Junior LLC, Moscow, Russia) was sterilized according to Simons et al. [40] and incubated overnight at 4 °C for emergency germination. The pre-germinate seeds were inoculated for 10 min in a fungal spore suspension (spore diluted in 1% carboxymethyl cellulose to 10^3^ CFU/mL, prepared as previously described by Afordoanyi et al. [41]) of each isolated strain and planted in pots (34.0 × 16.0 × 13.0 cm) containing sand amended with plant nutrient solution (PNS) [42]. The control group was prepared under the same conditions but without pathogens. Pots were incubated in a climate-controlled chamber under the following conditions: light intensity, 90%; day–night cycles, 16:8; humidity, 70%; and temperature, 26 ± 1 °C. For statistical analysis, 50 seeds inoculated with each *Fusarium* strain suspension were planted in a pot (a sample group). Each sample group was maintained in three replicates. For statistical analysis, the experiment was repeated in three independent tests. After 30 days of incubation, the plants were examined. The pathogenicity score (P.S.) of each isolated fungal strain was calculated using the following formula [43]:

$$P.S=\left(\sum \frac{ab}{NK}\right)\times 100$$

where ∑ ab represents the total obtained by multiplying the number of diseased plants by their corresponding degree of damage. “N” is the total number of plants analyzed, representing the highest grade on the scale. This scale ranges from 0 to 4, where 0 indicates asymptomatic plants, 1 represents plants with small lesions (<2 mm), 2 indicates moderate damage without severe harm to the plant, 3 represents severe damage or developed lesions, and 4 indicates dead plants.

#### 2.3.3. Pathogenicity Assay of Onion Bulbs

Koch’s postulates were used to confirm the pathogenicity of the isolated *Fusarium* species. For this purpose, onion bulbs were injected with 25 μL of *Fusarium* spore suspension (spore diluted in distilled water to 10^3^ CFU/mL) using a sterile syringe. For the control group, onion bulbs were injected with sterile distilled water. Onion bulbs were placed in a container (34.0 × 16.0 × 13.0 cm) and incubated in the dark for up to 3 weeks at 24 ± 1 °C. For each treatment, 10 onion bulbs were maintained in 3 independent replicates over time. After incubation, onion bulbs were cut with a scalpel, and the severity of symptoms of *Fusarium* was evaluated as follows: no visible symptoms (0), slight damage (1), moderate damage (2), severe damage (3), high damage and bulb death (4) according to Tirado-Ramirez et al. [44]. After 4 weeks, onion bulbs were examined using the following formula, as described in Section 2.3.1.

#### 2.3.4. In Vitro Control of Fusarium Isolates Using *Bacillus velezensis* KS04-AU and *Streptomyces albidoflavus* MGMM6

The antagonistic effects of *B. velezensis* KS04-AU (bacterial strain isolated from *Senna occidentalis* [45], microbial collection of FRC Kazan Scientific Center) and *S. albidoflavus* MGMM6 (bacterial strain isolated from the rhizosphere soil of spring wheat (*Triticum aestivum* L.) [46], microbial collection of FRC Kazan Scientific Center) against isolated *Fusarium* strains were assayed in dual cultures on PDA media. For this purpose, 10 µL of the highly virulent fungal strain suspension prepared in Section 2.3.1 was inoculated in the center of agar plates, after which 5 µL of the cell suspension of the KS04-AU and MGMM6 bacterial strains was co-inoculated. The plates were incubated at 28 ± 1 °C for up to 10 days.

### 2.4. In Planta Control of Fusarium Isolates through Bacillus velezensis KS04-AU and Streptomyces albidoflavus MGMM6

The in planta control of *Fusarium* isolates associated with onion bulbs through S. albidoflavus MGMM6 was evaluated as described in Section 2.3.1. For this purpose, non-sterile seeds were inoculated in the bacterial suspension of *S. albidoflavus* MGMM6 for 15 min and dried in a Laminar hood. Seeds were then planted in pots filled with sand mixed with a suspension of fungal spores (a consortium of pathogenic isolated *Fusarium* strains) diluted in PNS to a final concentration of 10^3^ CFU/mL. For the control group, seeds were treated with water. For statistical analysis, 50 seeds inoculated with each Fusarium strain suspension were planted in a pot (sample group). Each sample group was maintained in three replicates. After 4 weeks, the ability of *B. velezensis* KS04-AU and *S. albidoflavus* MGMM6 to suppress disease-caused *Fusarium* strains was examined as prescribed in Section 2.3.1.

### 2.5. Statistical Analysis

Statistical data analysis was performed using the statistical program OriginLab Pro SR1 b9.5.1.195 (OriginLab Corp., Northampton, MA, USA). Disease development between groups was analyzed using one-way ANOVA and post hoc Tukey’s honestly significant difference test (*p* < 0.05). Pearson correlation analysis was used to evaluate the aggressiveness of the isolates towards onion plants and bulbs.

## 3. Results

### 3.1. Characterization and Identification of Fungal Isolates

**Figure 2:**
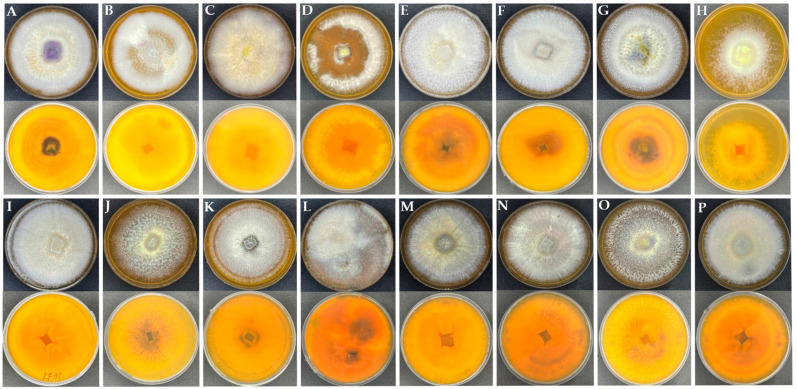
Phenotypical characteristics of *Fusarium* strains isolated from onion bulbs (*Allium cepa* L.). (**A**–**P**)—*Fusarium* isolated strains Fo1–Fo16, respectively. The isolated fungal strains were subsequently grown on Sabouraud media supplemented with rifampicin to a final concentration of 100 µg/µL.

The isolated fungal strains were subsequently grown on Sabouraud dextrose agar and examined under a microscope to assess their genus distribution based on colony and spore morphology. A total of 20 fungal strains were pre-isolated from onion bulbs, namely, Fo1 to Fo20. Visualization via microscopy (Figure S1) revealed that among the isolated strains, 16 belong to the large genus of filamentous fungi *Fusarium* (Figure 2).

### 3.2. BOX PCR for Genetic Diversity Analyses

**Figure 3:**
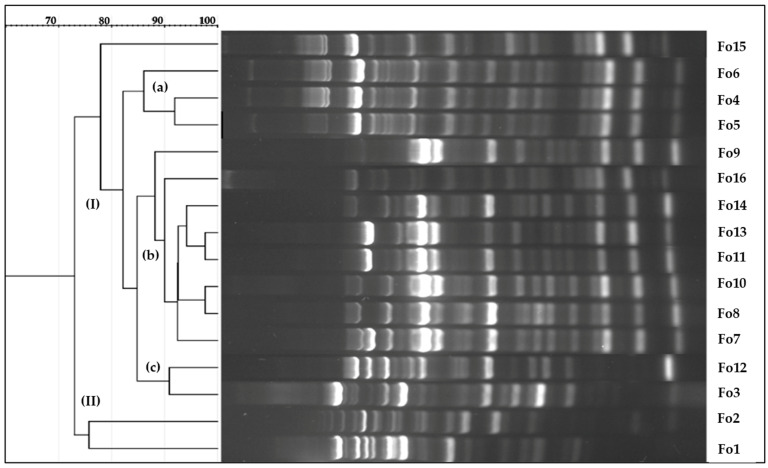
Dendrogram of BOX PCR constructed based on DNA profiles of fungal isolates using GelJ software version 3.0 [47]. Dendrograms were generated using the Jaccard similarity coefficient and UPGMA cluster analysis with a matching band tolerance set at 5%; (**I**) and (**II**) are major and minor clusters, respectively; (**a**–**c**) are out of subclusters.

Furthermore, to investigate the genetic diversity and discriminate the preselected strains, we performed BOX-PCR, and the results were analyzed using UPGMA. The results are shown in Figure 3. A dendrogram based on BOX elements revealed that the isolated strains could be classified into major and minor clusters (I and II) with a similarity coefficient of 90%. The major cluster included almost all the isolates, except for Fo1 and Fo2, which were included in a minor cluster. The major cluster comprises three subclusters (a, b, c) with a similarity coefficient of more than 90%. In addition, an out subcluster was formed among isolates Fo8 and Fo10, Fo4 and Fo5, and Fo11 and Fo13, all of which exhibited up to 97% coefficient similarities, indicating a high level of genetic homogeneity.

### 3.3. Molecular Identification

The molecular identification of *Fusarium* isolates at the strain level was confirmed using ITS and tef-1α. BLAST search for similar ITS partial genes and those present in the NCBI GenBank database showed that all the isolated strains belonged to the *Fusarium* genus, with similarity percentages ranging from 93% to 98% (Table 1). The obtained tef-1α sequences were analyzed using the Fusarium-ID database. The isolated strain Fo1 was identified as *F. oxysporum* with 96.95% similarity. The isolated strain Fo2, which belongs to the *Fusarium* complex group *Fusarium incarnatum-equiseti*, was identified as a *Fusarium* sp. with 96.60% similarity (Table 1). The isolated Fo6, Fo7, and Fo16 strains, which belong to the *F. fujikuroi* species complex (GFSC), were identified as *F. proliferatum* Fo3, Fo4, Fo5, and other isolated strains belonging to *Fusarium fujikuroi* were identified as *F. proliferatum*, with similarity percentages ranging from 93.02% to 97.67%. The amplicons of ITS gene fragments of the isolated *Fusarium* species were further deposited in the NCBI GenBank database.

**Figure 4:**
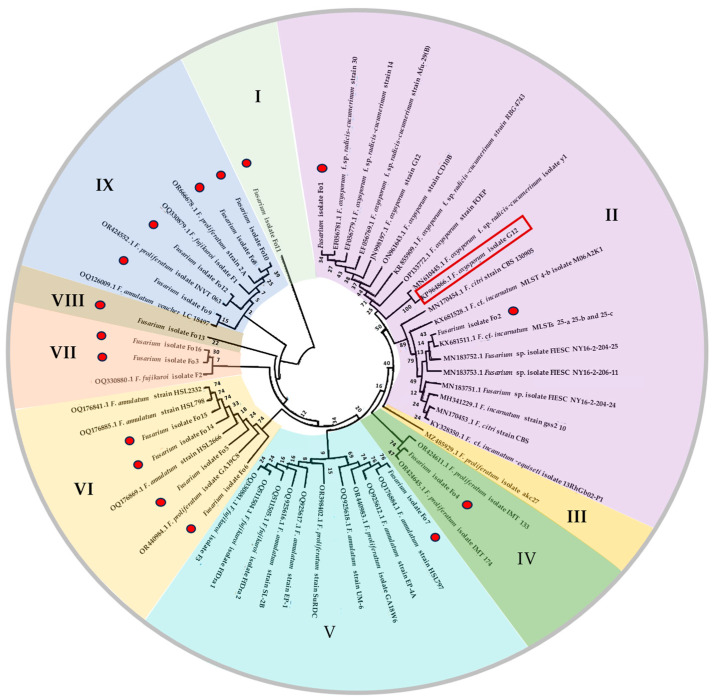
Maximum likelihood tree of 16 isolated *Fusarium* species based on the tef-α genes partial (Supplementary Material and Data). Bootstrap values (1000 replicates) are indicated on the branches. *Fusarium oxysporum* isolate G12 used as the outgroup for rooting the generated tree is highlighted with a red box. The fungal strains isolated in this study are marked with a red dot. I–IX as the number of generated clusters.

A phylogenetic tree analysis based on the neighbor-joining (NJ) method was performed to identify the phylogenetic relationships of the isolated *Fusarium* isolates. The *F. oxysporum* isolate G12 was used as the outgroup for rooting the gene tree. The analysis showed that the isolated fungal strains could be grouped into nine major clusters with significant variability in bootstrap frequency (Figure 4). The analysis revealed the wide genetic diversity of the isolated strains. *Fusarium oxysporum* Fo1 and *Fusarium* sp. Fo2 were grouped in cluster II and found to be closely related to *F. oxysporum*. The isolated fungal strains Fo4, Fo6, Fo14, and Fo15, which were grouped in cluster IV, were found to be most closely related to the species *F. proliferatum*. *Fusarium* strain Fo4, which was grouped in cluster IV, was found to be the closest related to *F. proliferatum*. *Fusarium* strain Fo7 formed cluster V with *F. annulatum* strain HSL797, whereas strains Fo6, Fo5, Fo14, and Fo15 were grouped in cluster VI with other strains of *F. annulatum* and *F. proliferatum*. *Fusarium* strains Fo3 and Fo16 formed cluster VII with *F. fujikuroi* isolate F2. The isolated fungal strains Fo13 and Fo11, grouped in clusters VIII and I, respectively, were found to constitute an outgroup cluster. The isolated fungal strains Fo10, Fo8, Fo12, and Fo9 identified as *F. proliferatum* were grouped in cluster IX with other strains of *F. annulatum* voucher LC 18497, *F. fujikuroi* isolate F1, *F. proliferatum* strain 2A, and *F. proliferatum* isolate INVT 063.

### 3.4. Pathogenicity Assay of Onion Seeds and Planta Control Using Bacillus velezensis KS04-AU

#### 3.4.1. Pathogenicity Assay of Onion Seeds

**Figure 5:**
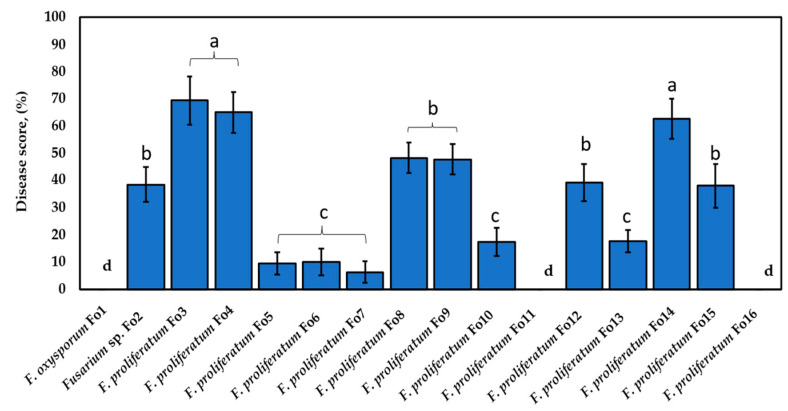
Pathogenicity of *Fusarium* strain on onion seeds. Different letters above the bars indicate a significant difference at *p* < 0.05 according to ANOVA and Tukey’s post hoc tests.

Pathogenicity tests were also conducted using a subset of the genetic diversity generated by BOX-PCR (Figure 3). The obtained results are shown in Figure 5. Nearly all the isolated fungal strains were found to be pathogenic to onions. However, exceptions were observed for *F. oxysporum* Fo1, *F. proliferatum* Fo11, and Fo16, which did not cause any disease symptoms, such as necrosis in the radicle or leaf curling on plant onion seedlings. The aggressiveness of the isolated strains could be classified into three categories: high, intermediate, and low aggressive fungi. The isolates Fo3, Fo4, and Fo14 were found to be highly pathogenic to onion plants. The aggressive indices of the two groups were calculated to be 69.37 ± 8.81%, 64.97 ± 7.42%, and 62.61 ± 7.25%, respectively (Figure 3). In addition, no significant difference between these isolates was observed at *p* < 0.05.

Pretreated plants with *Fusarium* sp. Fo2 and *F. proliferatum* Fo8, Fo9, Fo12, or Fo15 exhibited intermediate aggressiveness, with no significant difference (*p*-value < 0.05). The aggressive indices were 38.51 ± 6.39%, 48.23 ± 5.59%, 47.77 ± 5.71%, 39.20 ± 6.91%, and 38.01 ± 7.97%, respectively. Finally, *F. proliferatum* Fo5, Fo6, Fo7, Fo10, and Fo13 exhibited up to 3.5-fold lower disease indices than did the highly virulent fungal isolates Fo3, Fo4, and Fo14. In line with this, no significant difference in aggressivity was observed between isolates Fo7 and Fo10. Additionally, strains Fo1, Fo11, and Fo16 were found to be aggressive toward seedling onions.

#### 3.4.2. Pathogenicity Assay of Onion Bulbs

**Figure 6:**
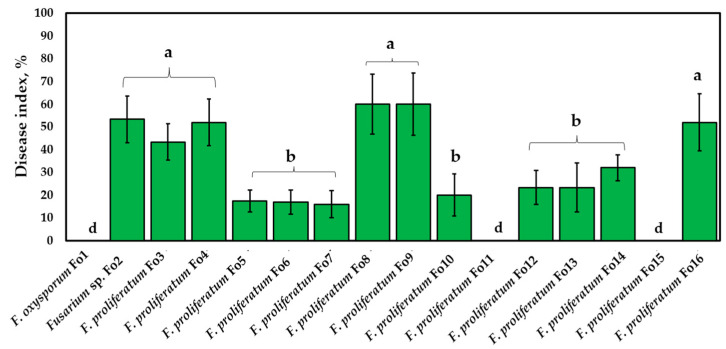
Pathogenicity of isolated *Fusarium* strains in bulb onions (*Allium cepa* L.). Koch’s postulates were applied in three replicates, and the test was repeated twice. Different letters above the bars indicate a significant difference at *p* < 0.05 according to ANOVA and Tukey’s post hoc tests.

The results revealed that the isolated *Fusarium* strains could be classified into two categories according to their aggressiveness (intermediate and low-virulence fungi) (Figure 6).

**Figure 7:**
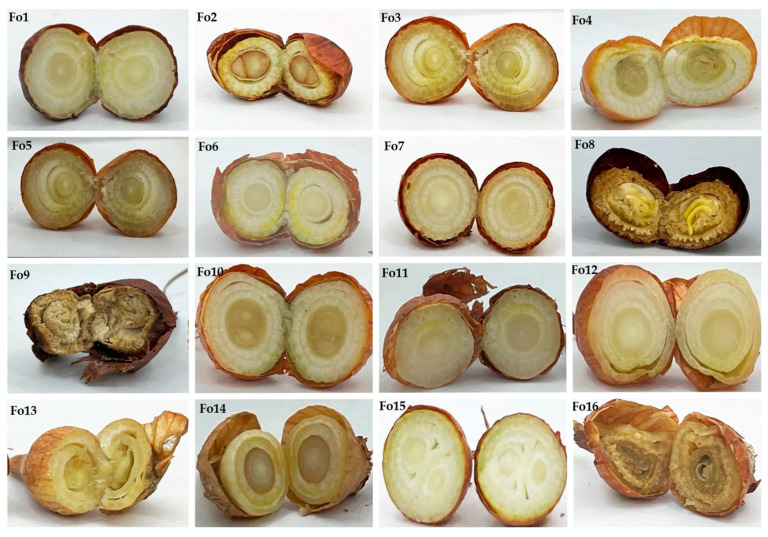
Koch postulates analysis of isolated *Fusarium* strains.

According to Koch’s postulate, the Fo2, Fo3, Fo4, Fo8, Fo9, and Fo16 strains exhibited high aggression toward onion bulbs, but the differences were not significant (*p* < 0.05). These strains cause typical symptoms of *Fusarium* infection in onions, such as rotting of bulbs and whitish mycelium on the bulb surface (Figure 7). The disease indices were 53.33 ± 10.32%, 43.41 ± 8.04%, 52.04 ± 9.87%, 60.15 ± 10.53%, 60.24 ± 11.08%, and 52.04 ± 12.43%, respectively. In contrast, the *Fusarium* strains Fo5, Fo6, Fo7, Fo10, Fo12, Fo13, and Fo14 exhibited low aggressiveness, ranging between 16% and 32%, without statistical significance (*p*-value < 0.05). Their aggressivities were 17.5 ± 4.8%, 16.9 ± 5.23%, 16.01 ± 5.87%, 20.05 ± 9.21%, 23.36 ± 10.73%, 23.36 ± 9.73%, and 32.06 ± 5.78%, respectively. Moreover, *F. oxysporum* Fo1, Fo11, and Fo15 were found to be unaggressive toward the onion bulbs used in this study.

### 3.5. In Vitro Control of Fusarium Isolates Using Bacillus velezensis KS04-AU and S. albidoflavus MGMM6

**Figure 8:**
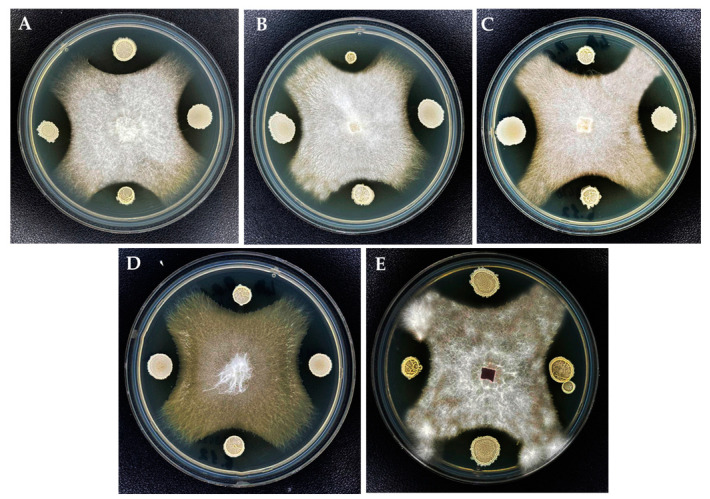
Antagonistic activity of *B. velezensis* KS04-AU (bacterial strain inoculated at the left and right of the growing fungus) and *S. albidoflavus* MGMM6 (bacterial strain inoculated above and below the growing fungus) against *Fusarium* strains isolated from onion bulbs (*Allium cepa* L.). *F. oxysporum* Fo1 (**A**), *Fusarium* sp. Fo2 (**B**), *F. proliferatum* Fo3 (**C**), *F. proliferatum* Fo9 (**D**), and *Fusarium proliferatum* Fo13 (**E**).

For qualitative antagonistic analysis, the strains were selected based on their aggressiveness towards plants and onion bulbs. Five strains were further chosen: one non-aggressive strain, two highly aggressive strains, and two low aggressive *Fusarium* strains. The antagonistic effect of *B. velezensis* KS04-AU and *S. albidoflavus* MGMM6 against *Fusarium* strains is shown in Figure 8. Both *B. velezensis* KS04-AU and *S. albidoflavus* MGMM6 exhibited moderate inhibitory activity against the tested phytopathogenic fungi *F. oxysporum* Fo1 (A), *Fusarium* sp. Fo2 (B), *F. proliferatum* Fo3 (C), *F. proliferatum* Fo9 (D), and *Fusarium proliferatum* Fo13 (E). In addition, among the tested phytopathogenic fungi *F. oxysporum* Fo1, *Fusarium* sp. Fo2, *F. proliferatum* Fo3, and *F. proliferatum* Fo9 were strongly suppressed by KS04-AU.

### 3.6. In Planta Control of Fusarium Isolates through Bacillus velezensis KS04-AU and Streptomyces albidoflavus MGMM6

**Figure 9:**
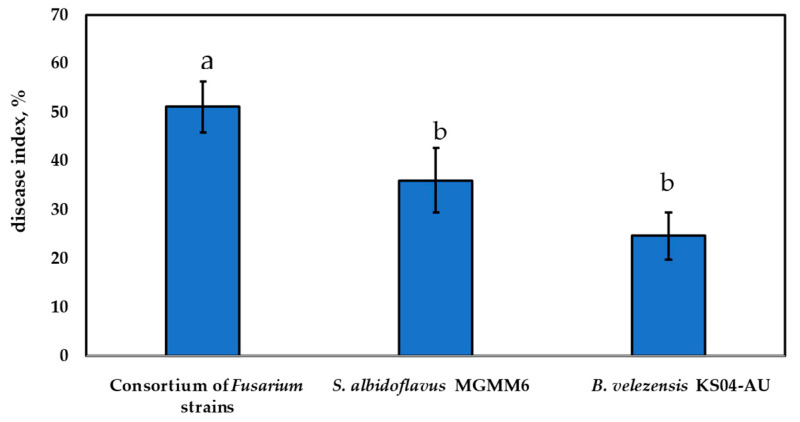
Biocontrol ability of *B. velezensis* KS04-AU and *S. albidoflavus* MGMM6 against a consortium of isolated *Fusarium* strains. Different letters above the bars indicate a significant difference at *p* < 0.05 according to ANOVA and Tukey’s post hoc tests.

The ability of *S. albidoflavus* MGMM6 and *B. velezensis* KS04-AU in planta to protect onion plants and inhibit the growth of a consortium of *Fusarium* strains isolated in this study is shown in Figure 9. The most observed symptoms were necrosis in the radicle, apical strangulation, and leaf curling. The results demonstrated a significant decrease in disease development at a *p*-value of *p* < 0.05. The disease index in the group of plants pretreated with *S. albidoflavus* MGMM6 and *B. velezensis* KS04-AU was up to 12.64 ± 3.45% and 18.65 ± 4.26%, respectively, less than those without treatment (treated with a consortium of pathogenic *Fusarium* strains), which was assessed as 51.17 ± 5.22%. The effectiveness of *S. albidoflavus* MGMM6 and *B. velezensis* KS04-AU against these phytopathogens was measured as 24.73 ± 4.86% and 36.06 ± 6.61%, respectively.

**Figure 10:**
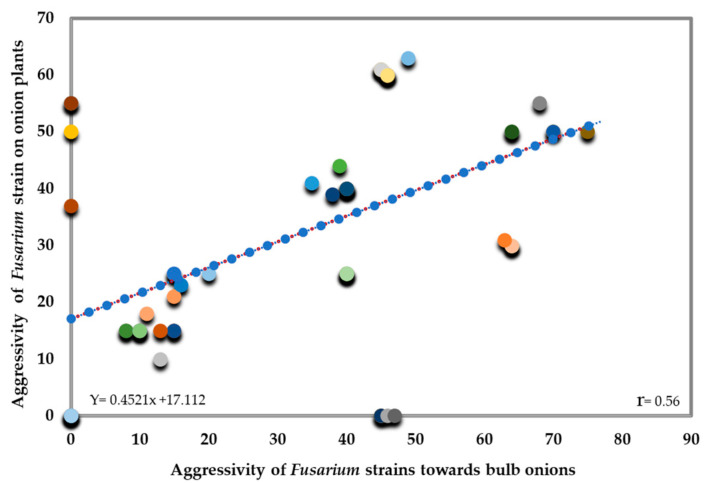
Correlation between the aggressivity of isolated *Fusarium* strains towards onion bulbs and on plants. Each data point represents a relationship between aggressivity of isolated *Fusarium* strains.

In terms of their aggressiveness towards onion bulbs and plants, a moderate positive correlation was observed (R = 0.566, *p* < 0.01) (Figure 10). This suggests that as the aggressiveness of strains increases, there is a moderate tendency to increase damage to both onion bulbs and plants, indicating a constant but not ideal relationship between the aggressiveness of strains and the harm they cause to onion bulbs and plants.

## 4. Discussion

*Fusarium*, a prominent genus of filamentous fungi, encompasses numerous plant pathogenic species that cause significant agricultural yield losses, reaching up to 14% annually [47,48,49,50,51]. Therefore, studying the diversity of these organisms is crucial for understanding their interactions in ecosystems and developing effective strategies for disease control, crop protection, and ecosystem conservation. Plant pathogens exhibit rapid and continuous evolutionary changes in response to various environmental pressures, including climate change and disease control measures. These stresses on pathogens necessitate constant monitoring and understanding to effectively manage and mitigate their impact on plants and ecosystems [52,53,54,55,56]. In this study, we characterized the diversity of fungal strains that cause spoilage of onion bulbs. We found that this spoilage was primarily caused by *Fusarium* species. To confirm the genetic diversity of the isolated fungal species, we used BOX-PCR, a well-known molecular method that allows differentiation at the species level [57,58,59]. Molecular analysis of the *Fusarium* isolates using the tef gene revealed the strong presence of *F. proliferatum* (98%), *F. oxysporum* (1%), and *Fusarium* sp. (1%). Most of the isolated strains belonged to the *Fusarium fujikuroi* species complex, with 96% similarity and coverage. This complex is renowned for its ability to cause a range of destructive diseases in various crops, including onion [60,61,62,63,64,65,66]. Pathogenicity assessment is necessary to identify potential risks associated with the introduction of virulent species or strains into new geographical regions or crops. Koch’s postulate, based on measuring the pathogenicity of isolated pathogenic strains via inoculation, followed by monitoring seedling emergence, survival, and health [67,68,69,70,71,72], is a crucial method for evaluating and developing effective disease control strategies, as well as resistance to these pathogens, in various plant varieties. Koch’s postulates on onion seeds and bulbs confirmed that the isolates developed different disease indices, reaching up to 96% with wilt symptoms such as discoloration, wilting, stunted growth, and spoilage of the onion bulbs. The diversity of pathogenicity of these isolated strains in infecting onion seedlings and bulbs may be related to their lifestyle, such as the secretion of pathogenicity-related genes, infection mechanisms, and interactions with plant hosts [73,74]. Interestingly, the nonaggressive *Fusarium* strains isolated in this study belonged to a subgroup phylogenetically distinct from highly and moderately aggressive pathogenic strains. These findings suggested that these isolates are forma specialis to onions, as previously reported by Dissanayake et al. [75] and Sasaki et al. [76]. The genus *Fusarium* contains a variety of complex species, some of which are non-pathogenic and pathogenic depending on the host plant. Non-pathogenic *Fusarium* species such as *F. oxysporum* have been identified as biocontrol agents and endophytic agents [77,78]. Moreover, these non-pathogenic *Fusarium* strains that have been isolated from onion may proliferate and become pathogenic to other plant species through crop rotation, making them difficult to control once they have contaminated crops. This difficulty arises because of the lack of reliable morphological features in the *Fusarium* complex, leading to the indistinguishability of pathogenic and non-pathogenic isolates. Therefore, the determination of pathogenicity still relies on tests conducted on host plants.

According to Koch’s postulation of seedlings, the development of disease symptoms in isolated *Fusarium* strains tends to be slower than that in onion bulbs. Moreover, *Fusarium* strains that can infect onion plants were found to be less pathogenic to onion bulbs and vice versa. Similar results have been previously reported [79,80,81,82]. For example, a study conducted by Carrieri et al. [83] showed that *F. tricinctum* is harmful to onion plants, but Koch’s postulates did not cause any symptoms of disease in onion plants. The current methods of wilt disease control have several limitations. One promising alternative is the use of microorganisms to reduce disease development through biocontrol. Considering the pathogenic virulence diversity of the isolated strains, we wanted to develop an eco-friendly approach that could suppress the growth of these *Fusarium* strains, causing spoilage of onion bulbs during storage. Biocontrol analysis using *S. albidoflavus* MGMM6 and *B. velezensis* KS04-AU could control the growth of these pathogenic fungi in vitro. The use of a microbial agent to control the growth of phytopathogenic fungi during crop storage is considered a promising method for crop protection [84,85]. The effectiveness of *B. velezensis* and *S. albidoflavus* in inhibiting the growth of phytopathogens such as *Fusarium* species has been well documented [86,87]. Therefore, our results suggest that *S. albidoflavus* MGMM6 and *B. velezensis* KS04-AU are effective bioagents for suppressing the growth of isolated *Fusarium* strains. Several studies have demonstrated the ability of *S. albidoflavus* and *B. velezensis* isolates to suppress the growth of phytopathogenic fungi in planta [85,86,87,88]. In this study, we found that *S. albidoflavus* MGMM6 is less effective against a consortium of phytopathogenic *Fusarium* strains isolated from onion bulbs. This may be due to the strong pathogenicity induced by synergistic interactions of isolated *Fusarium* strains that contribute to their enhanced virulence when applied in a consortium, as reported by Sidharthan [89]. The synergistic interactions between these pathogenic strains may contribute to their collective virulence.

## 5. Conclusions

Phytopathogenic microorganisms can lead to significant crop losses, which comprise both qualitative damage that reduces the economic value of the crop and makes it unsuitable for human consumption. This study successfully identified and evaluated the aggressiveness of *Fusarium* species associated with onion bulbs during storage, shedding light on the potential threats to onion production and storage. The morphological and molecular differentiation of the isolated strains revealed their taxonomic identity belonging to *Fusarium* complex species *incarnatum-equiseti* and *Fusarium fujikuroi*. Koch’s postulate analysis demonstrated the varying aggressiveness of the isolated strains on onion bulbs and plants, with disease symptoms developing more slowly on plants according to the postulates. Importantly, this study highlighted the presence of *Fusarium* strains that could infect onion plants less aggressively than onion bulbs, or vice versa. In addition, some strains were found to be non-aggressive, which indicated the ability of each strain to occupy its ecological niche and did not suppress the viability of other fungi belonging to the same genus *Fusarium*. Moreover, we found that the development of isolated fungi can be controlled using a biological approach. This indicates the potential for developing an environmentally friendly alternative to controlling the growth of phytopathogens, which could lead to a reduction in onion losses during storage. This finding could serve as an instrument in the development of biocontrol strategies for onion crop protection against phytopathogenic microorganisms during storage. However, studies in field conditions with different onion varieties are needed to elucidate their potential effectiveness.

## Figures and Tables

**Table 1:** Molecular identification of fungal strains isolated from onion bulbs based on tef-1α and ITS genes.

Isolates	Marker	Species	Accession Number Deposited in NCBI Genbank	Similarity	Species Complex	Reference Accession Number
Fo1	tef-1α	*F. oxysporum*	PP140391	96.95%	*F. oxysporum*	FJ985420
	ITS	*F. oxysporum*		98.51%	*F. oxysporum*	DQ790536
Fo2	tef-1α	*Fusarium* sp.	PP140392	96.60%	*F. incarnatum-equiseti*	JF740715
	ITS			98.50%	*F. oxysporum*	DQ790536
Fo3	tef-1α	*F. proliferatum*	PP140393	97.67%	*F. fujikuroi*	MH582346
	ITS			98.00%	*F. oxysporum*	DQ790536
Fo4	tef-1α	*F. proliferatum*	PP140394	97.66%	*F. fujikuroi*	MH582346
	ITS			99.04%	*Gibberella fujikuroi*	U34569
Fo5	tef-1α	*F. proliferatum*	PP140395	97.30%	*F. fujikuroi*	MH582346
	ITS			98.57%	*Gibberella fujikuroi*	U34569
Fo6	tef-1α	*F. proliferatum*	PP140396	98.07%	*F. fujikuroi*	MH582347
	ITS			99.04%	*Gibberella fujikuroi*	U34569
Fo7	tef-1α	*F. proliferatum*	PP140397	95.80%	*F. fujikuroi*	MH582347
	ITS			98.56%	*Gibberella fujikuroi*	U34569
Fo8	tef-1α	*F. proliferatum*	PP140398	96.91%	*F. fujikuroi*	MH582346
	ITS			99.04%	*Gibberella fujikuroi*	U34569
Fo9	tef-1α	*F. proliferatum*	PP140399	94.02%	*F. fujikuroi*	MH582346
	ITS			97.00%	*F. incarnatum-equiseti*	DQ790541
Fo10	tef-1α	*F. proliferatum*	PP140400	95.74%	*F. fujikuroi*	MH582346
	ITS			99.04%	*Gibberella fujikuroi*	U34569
Fo11	tef-1α	*F. proliferatum*	PP140401	97.67%	*F. fujikuroi*	MH582346
	ITS			99.04%	*Gibberella fujikuroi*	U34569
Fo12	tef-1α	*F. proliferatum*	PP140402	95.38%	*F. fujikuroi*	MH582346
	ITS			98.56%	*Gibberella fujikuroi*	U34569
Fo13	tef-1α	*F. proliferatum*	PP140403	95.38%	*F. fujikuroi*	MH582346
	ITS			97.52%	*Gibberella fujikuroi*	U34569
Fo14	tef-1α	*F. proliferatum*	PP140404	95.38%	*F. fujikuroi*	MH582346
	ITS			98.61%	*Gibberella fujikuroi*	U34569
Fo15	tef-1α	*F. proliferatum*	PP140405	98.05%	*F. fujikuroi*	MH582346
	ITS			97.02%	*Gibberella fujikuroi*	U34569
Fo16	tef-1α	*F. proliferatum*	PP140406	98.07%	*F. fujikuroi*	MH582347
	ITS			98.54%	*Gibberella fujikuroi*	U34569

## Data Availability

Data are contained within the article and supplementary materials.

## Supplementary Materials

The following supporting information can be downloaded at: https://www.mdpi.com/article/10.3390/jof10020161/s1, Figure S1: Microscopic fungal spores’ visualization under light microscopy. Sequence data for the tef-1α gene partial from 16 *Fusarium* strains isolated from onion bulb (*Allium cepa* L.).
