# Supplementary material for: Identification and Aggressiveness of Fusarium Species Associated with Onion Bulb (Allium cepa L.) during Storage
Source: J Fungi (Basel). 2024 Feb 19;10(2):161. doi: 10.3390/jof10020161 (PMC10890437; doi:10.3390/jof10020161)
Supplement: Supplementary file 1 [file jof-10-00161-s001.zip › jof-2818131-supplementary.pdf]

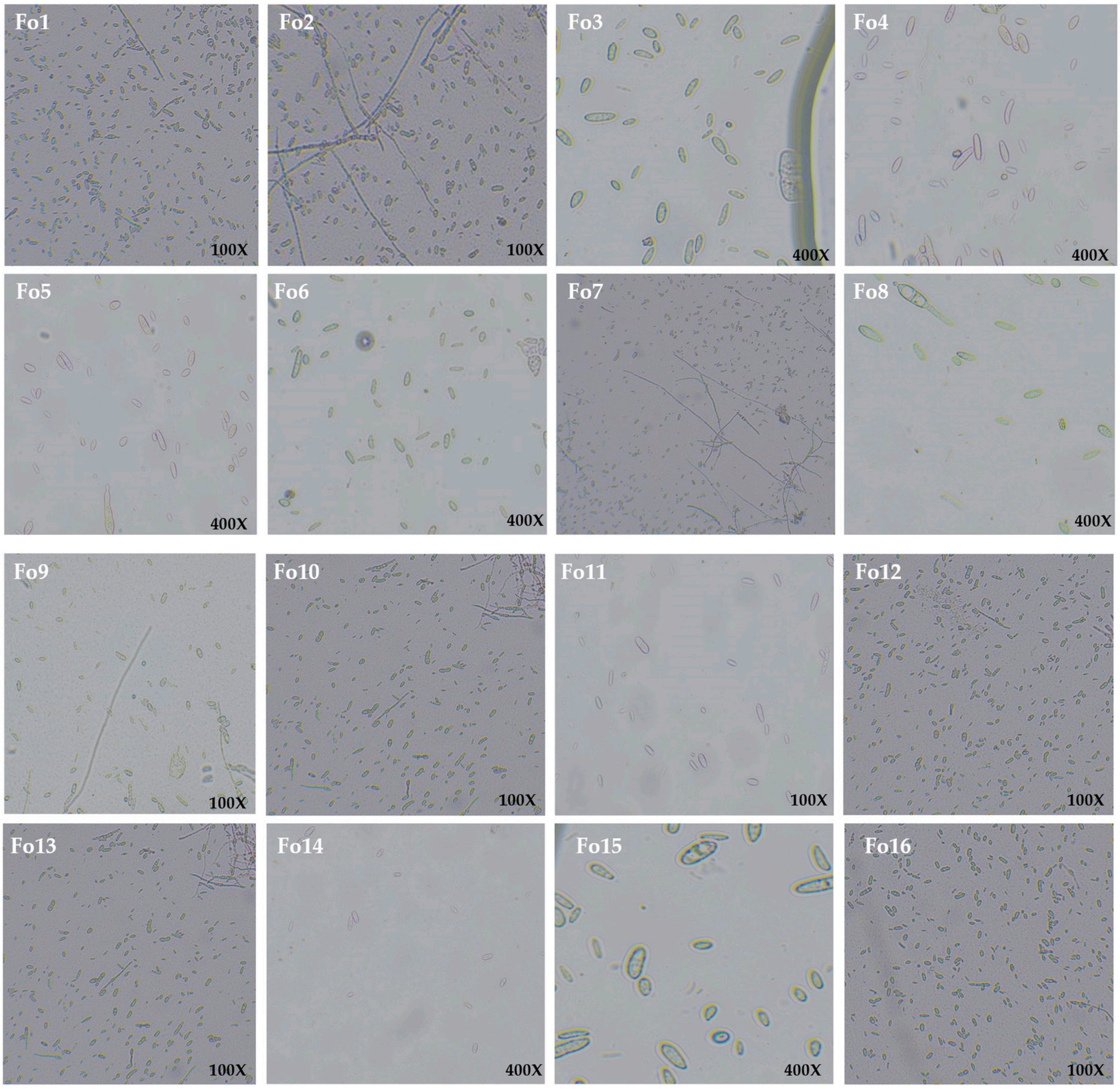

Figure S1. Microscopic fungal spores' visualization under light microscopy.

Sequence data for the tef-1α gene partial from 16 *Fusarium* strains isolated from onion bulb (*Allium cepa* L.)

>*Fusarium* isolate Fo1
TTTCTTCGATCGCGCGTCCTTTGCCCATCGATTTCCCCTACGACTCGAAACGTGCCCGCTACCCCGCTCGAGACCAA
AAATTTTGCAATATGATCGTAATTTTTTTGGTGGGGCACTTACCCCGCCACTTGAGCGACGGGAGCGTTTGCCCTCTT
AACCATTCTCACAACCTCAATGAGTGCGTCGTCACGTGTCAAGCAGTCACTAACCATTCAACAATAGGAAGCCGCT
GAGCTCGGTAAGGGTGCTTCAAGTAAAA

>*Fusarium* isolate Fo2
ATACTTCTCGATCGCGCGTCCTCTGCCCACCGATCCATCACCCGAATCCGTCTCACGACGACTGAATATGCGCCTGT
ACCCCGCTCGAGTACAAAATTTTGCGGTTCAATCGTAATTTTTTGGTGCGGCTTCTACCCCGCTACTCGAGTGACAG
GTGTTTGCCCTTTCCCACAAAATCATCTTGCGCATCACGTGTCAAACAGTCACTAACCACCCGACAATAGGAAGCC
GCCGAGCTCGGTAAGGGTC

>*F**usarium*** isolate Fo3
ATCATTGATCGCGCGTCCTCTGCCCACCGATTTCACTTGCGATTCGAAACGTGCCTGCTACCCCGCTCGAGACCAA
AAATTTTGCGATATGACCGTAATTTTTTTGGTGGGGCATTTACCCCGCCACTCGAGCGATGGGCGCGTTTTTGCCCTT
TCCTGTCCACAACCTCAATGAGCGCATTGTCACGTGTCAAGCAGCGACTAACCATTCGACAATAGGAAGCCGCTG
AGCTCGGTAAGGGTTCCTTCAAGTAAAA

>*Fusarium* isolate Fo4
TTCATTCGATCGCGCGTCCTCTGCCCACCGATTTCACTTGCGATTCGAAACGTGCCTGCTACCCCGCTCGAGACCAA
AAATTTTGCGATATGACCGTAATTTTTTTGGTGGGGCATTTACCCCGCCACTCGAGCGATGGGCGCGTTTTTGCCCTT
TCCTGTCCACAACCTCAATGAGCGCATTGTCACGTGTCAAGCAACGACTAACCATTCGACAATAGGAAGCCGCTG
AGCTCGGTAAGGGTCCCTTCAAGTAA

>*Fusarium* isolate Fo5
TTATTCGATCGCGCGTCCTCTGCCCACCGATTTCACTTGCGATTCGAAACGTGCCTGCTACCCCGCTCGAGACCAAA
AATTTTGCGATATGACCGTAATTTTTTTGGTGGGGCATTTACCCCGCCACTCGAGCGATGGGCGCGTTTTTGCCCTTT
CCTGTCCACAACCTCAATGAGCGCATTGTCACGTGTCAAGCAACGACTAACCATTCGACAATAGGAAGCCGCTGA
GCTCGGTAAGGGTCCCTTCAAGTAAAA

>*Fusarium* isolate Fo6
TTTCATCGATCGCGCGTCCTCTGCCCACCGATTTCACTTGCGATTCGAAACGTGCCTGCTACCCCGCTCGAGACCAA
AAATTTTGCGATATGACCGTAATTTTTTTGGTGGGGCATTTACCCCGCCACTCGAGCGATGGGCGCGTTTTTGCCCTT
TCCTGTCCACAACCTCAATGAGCGCATTGTCACGTGTCAAGCAGCGACTAACCATTCGACAATAGGAAGCCGCTG
AGCTCGGTAAGGGTTCCCTTCAAGTAA

>*Fusarium* isolate Fo7
TTTCTTCGATCGCGCGTCCTCTGCCCACCGATTTCACTTGCGATTCGAAACGTGCCTGCTACCCCGCTCGAGACCAA
AAATTTTGCGATATGACCGTAATTTTTTTGGTGGGGCATTTACCCCGCCACTCGAGCGATGGGCGCGTTTTTGCCCTT
TCCTGTCCACAACCTCAATGAGCGCATTGTCACGTGTCAAGCAGCGACTAACCATTCGACAATAGGAAGCCGCTG
AGCTCGGTAAGTTCCTTCAAGTAAAATA

>*Fusarium* isolate Fo8
TTCTTCGATCGCGCGTCCTCTGCCCACCGATTTCACTTGCGATTCGAAACGTGCCTGCTACCCCGCTCGAGACCAAA
AATTTTGCGATATGACCGTAATTTTTTTGGTGGGGCATTTACCCCGCCACTCGAGCGATGGGCGCGTTTTTGCCCTTT
CCTGTCCACAACCTCAATGAGCGCATTGTCACGTGTCAAGCAGCGACTAACCATTCGACAATAGGAAGCCGCTGA
GCTCGGTAAGGGCCCTTCAAGTAAGA

>*Fusarium* isolate Fo9
TTCTTCGATCGCGCGTCCTCTGCCCACCGATTTCACTTGCGATTCGAAACGTGCCTGCTACCCCGCTCGAGACCAAA
AATTTTGCGATATGACCGTAATTTTTTTGGTGGGGCATTTACCCCGCCACTCGAGCGATGGGCGCGTTTTTGCCCTTT
CCTGTCCACAACCTCAATGAGCGCATTGTCACGTGTCAAGCAGCGACTAACCATTCGACAATAGGAAGCCGCTGA
GCTCGGTAAGGTGGATTCAAGTAAAA

>*Fusarium* isolate Fo10
TTCCTTCGATCGCGCGTCCTCTGCCCACCGATTTCACTTGCGATTCGAAACGTGCCTGCTACCCCGCTCGAGACCAA
AAATTTTGCGATATGACCGTAATTTTTTTGGTGGGGCATTTACCCCGCCACTCGAGCGATGGGCGCGTTTTTGCCCTT
TCCTGTCCACAACCTCAATGAGCGCATTGTCACGTGTCAAGCAACGACTAACCATTCGACAATAGGAAGCCGCTG
AGCTCGGTAAGTTCCATTCAAGTAAA

>*Fusarium* isolate Fo11
TTATTCGATCGCGCGTCCTCTGCCCACCGATTTCACTTGCGATTCGAAACGTGCCTGCTACCCCGCTCGAGACCAAA
AATTTTGCGATATGACCGTAATTTTTTTGGTGGGGCATTTACCCCGCCACTCGAGCGATGGGCGCGTTTTTGCCCTTT
CCTGTCCACAACCTCAATGAGCGCATTGTCACGTGTCAAGCAGCGACTAACCATTCGACAATAGGAAGCCGCTGA
GCTCGGTAAGGGCCCTTCAAGTAA

>*Fusarium* isolate Fo12
TTCCTTCGATCGCGCGTCCTCTGCCCACCGATTTCACTTGCGATTCGAAACGTGCCTGCTACCCCGCTCGAGACCAA
AAATTTTGCGATATGACCGTAATTTTTTTGGTGGGGCATTTACCCCGCCACTCGAGCGATGGGCGCGTTTTTGCCCTT
TCCTGTCCACAACCTCAATGAGCGCATTGTCACGTGTCAAGCAGCGACTAACCATTCGACAATAGGAAGCCGCTG
AGCTCGGTAAGGTCAATTCAAGTAAAGT

>*Fusarium* isolate Fo13
TTTCTTCGATCGCGCGTCCTCTGCCCACCGATTTCACTTGCGATTCGAAACGTGCCTGCTACCCCGCTCGAGACCAA
AAATTTTGCGATATGACCGTAATTTTTTTGGTGGGGCATTTACCCCGCCACTCGAGCGATGGGCGCGTTTTTGCCCTT
TCCTGTCCACAACCTCAATGAGCGCATTGTCACGTGTCAAGCAGCGACTAACCATTCGACAATAGGAAGCCGCTG
AGCTCGGTAAGGGTCCCTTCAAGTAA

>*Fusarium* isolate Fo14
TTATTCGATCGCGCGTCCTCTGCCCACCGATTTCACTTGCGATTCGAAACGTGCCTGCTACCCCGCTCGAGACCAAA
AATTTTGCGATATGACCGTAATTTTTTTGGTGGGGCATTTACCCCGCCACTCGAGCGATGGGCGCGTTTTTGCCCTTT
CCTGTCCACAACCTCAATGAGCGCATTGTCACGTGTCAAGCAGCGACTAACCATTCGACAATAGGAAGCCGCTGA
GCTCGGTAAGGGTAACTTCAAGTAA

>*Fusarium* isolate Fo15
TTCGTTCGATCGCGCGTCCTCTGCCCACCGATTTCACTTGCGATTCGAAACGTGCCTGCTACCCCGCTCGAGACCAA
AAATTTTGCGATATGACCGTAATTTTTTTGGTGGGGCATTTACCCCGCCACTCGAGCGATGGGCGCGTTTTTGCCCTT
TCCTGTCCACAACCTCAATGAGCGCATTGTCACGTGTCAAGCAGCGACTAACCATTCGACAATAGGAAGCCGCTG
AGCTCGGTAAGGGTCCCTTCAAGTAA

>*Fusarium* isolate Fo16
CTTTGATCGCGCGTCCTCTGCCCCCGATTTCACTTGCGATTCGAAACGTGCCTGCTACCCCGCTCGAGACCAAAAAT
TTTGCGATATGACCGTAATTTTTTTGGTGGGGCATTTACCCCGCCACTCGAGCGATGGGCGCGTTTTTGCCCTTTCCT
GTCCACAACCTCAATGAGCGCATTGTCACGTGTCAAGCAGCGACTAACCATTCGACAATAGGAAGCCGCTGAGCT
CGGTAAGGGTTCCTTCAAGTAA
